# Detection of mixed-strain infections by FACS and ultra-low input genome sequencing

**DOI:** 10.1080/19490976.2018.1526578

**Published:** 2018-10-05

**Authors:** Mária Džunková, Andrés Moya, Xinhua Chen, Ciaran Kelly, Giuseppe D’Auria

**Affiliations:** a Department of Genomics and Health, Foundation for the Promotion of Health and Biomedical Research of Valencia Region (FISABIO-Public Health), València, Spain; b CIBER in Epidemiology and Public Health (CIBEResp), Madrid, Spain; c Institute for Integrative Systems Biology (I2SysBio), The University of Valencia and The Spanish National Research Council (CSIC)-UVEG), València, Spain; d Australian Centre for Ecogenomics, The University of Queensland, St Lucia, Australia; e Division of Gastroenterology, Beth Israel Deaconess Medical Center, Harvard Medical School, Boston, USA; f Sequencing and Bioinformatics Service of the Foundation for the Promotion of Health and Biomedical Research of Valencia Region (FISABIO-Public Health), València, Spain

**Keywords:** FACS-seq, *C. difficile*, mixed-strain infection, low-input DNA sequencing, epidemiology

## Abstract

The epidemiological tracking of a bacterial outbreak may be jeopardized by the presence of multiple pathogenic strains in one patient. Nevertheless, this fact is not considered in most of the epidemiological studies and only one colony per patient is sequenced. On the other hand, the routine whole genome sequencing of many isolates from each patient would be costly and unnecessary, because the number of strains in a patient is never known *a priori*. In addition, the result would be biased by microbial culture conditions.

Herein we propose an approach for detecting mixed-strain infection, providing *C. difficile* infection as an example. The cells of the target pathogenic species are collected from the bacterial suspension by the fluorescence activated cell sorting (FACS) and a shallow genome sequencing is performed. A modified sequencing library preparation protocol for low-input DNA samples can be used for low prevalence gut pathogens (< 0.1% of the total microbiome). This FACS-seq approach reduces diagnostics time (no culture is needed) and may promote discoveries of novel strains. Methodological details, possible issues and future directions for the sequencing of these natural pan-genomes are herein discussed.

## Introduction

Whole genome sequencing technologies have continuously become more efficient, and thus they have the potential to become a common diagnostic tool allowing accurate identification of pathogenic strains. The most habitual approach in the epidemiological studies is to sequence one colony per patient. However, recent studies showed that if only one colony per patient is screened, only as few as 25 % of cases can be linked to the previously isolated strains within the same hospital.^1-3^ This discrepancy is caused by the presence of multiple strains in a single patient. These mixed-strain infections can impair the exclusion of transmission and determination of the outbreak origin.

As the number of strains in a patient is never known *a priori*, the routine whole genome sequencing of many isolates from each patient would be costly and unnecessary. In addition, as different strains often have very different growth requirements, it may be impossible to isolate all the different strains from one patient. One option for obtaining genome sequences of all strains in their natural proportion without culture would be the metagenomic sequencing of the biological sample. However, such an approach would not be feasible for pathogens which form very small portion of the total microbiome.

*Clostridium difficile* infection (CDI) is one of the numerous diseases for which co-infection by multiple strains is often reported.^4-6^ CDI has nosocomial origin and its severity and mortality is increasing.^7^ *Clostridium difficile*, recently renamed *Clostridioides difficile*,^8^ forms only 0.0001–0.1 % of the infected microbiome.^9,10^ Its strains have different growth rate and culture medium requirements.^11^ As the isolation of *C. difficile* is usually done with a single culture media, the mixed-strain infections often remain undetected.

Recovery of multiple *C. difficile* genomes directly from the metagenomic sequences would be very difficult because of its low proportion in the gut microbiome. However, the fluorescence activated cell sorting (FACS) can be used for enrichment of the target bacterial species prior to sequencing.^12^ While this FACS-seq method is mostly used for genomic characterization of uncultured bacteria, it has not been used in clinical microbiology yet. However, FACS-seq can have high importance in studies focused on low-prevalent pathogenic bacterial species whose strains have very different culture requirements.

### Facs-seq of *C. difficile* cells

As an example for this commentary/view article, we performed the FACS-seq of *C. difficile* with one faecal sample from a patient who was hospitalized in May 2014 at the Beth Israel Deaconess Medical Center of the Harvard Medical School, Boston, MA, USA. The patient was diagnosed to be CDI positive by routine Illumigene assay (Meridian Biosiences). The study was approved by the institutional review board of BIDMC, and a written informed consent was obtained from the participant. The proportion of *C. difficile* in the patient’s faecal sample was 0.1% (quantified by Qiagen qPCR kit # BPID00110AF targeting *C. difficile* specific 16S rDNA sequences).

**Figure 1.**
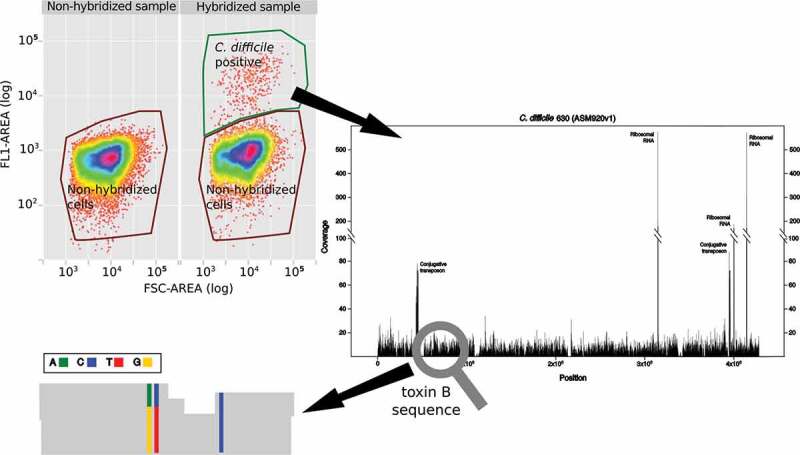
Work-flow chart of the detection of mixed-strain infections by FACS and ultra-low input genome sequencing.

The 16S rDNA probes were used for hybridization of *C. difficile* cells present in the feacal bacterial suspension as described by Novakova *et al*.^13^ A sample containing 90 % of faecal bacteria from the patient and 10% of cultured *C. difficile* cells (ATCC 9689) was used as an additional FACS sorting control. The FACS was performed on S3 cell sorter (Bio-Rad). The first cell selection gate was set on the red 640 nm channel (FL4) to discard organic particles present in faeces which are not fluorescent when stained by the DNA stain SYTO 62. The cells containing DNA were then visualized on the next bi-plots representing cell size (forward scatter) on the x-axis and green fluorescence on the FL1 channel (488 nm) on the y-axis (Figure 1). The final sorting gate was set by comparing the non-hybridized faecal samples with the *C. difficile* positive control.

The amount of extracted DNA from the 10,000 FACS-collected *C. difficile* cells was undetectable by Picogreen assay (Life Technologies). It means that the sample contained less DNA amount than the 50 ng required by the standard Illumina Nextera XT library preparation protocol version from the year 2014 (Ref. FC-121–1030) and it was also less than 1 ng required by the current version of the Nextera XT tagmentation protocol (Document number #15031942). Therefore, for the sequencing library preparation a modified protocol adjusted for ultra-low input DNA samples was used. The volume of the Nextera XT tagmentation mixture was reduced five times, while the volume of DNA sample was increased accordingly. The sample was sequenced with MiSeq® Reagent Kit v3.

The sequences have been deposited in European Nucleotide Archive database with study accession number PRJEB20472. Quality filtered reads were mapped to the *C. difficile* reference strain 630 (GenBank assembly accession GCA_000009205.1) by *Bowtie2* using the default parameters for “very-fast” mapping of only the most similar reads, requiring that the entire read align from one end to the other.^14^ The resulting mapping was inspected by the Integrative Genomics Viewer software.^15^ The coverage plots of the reference genome revealed that there was a high sequence variability in the areas of ribosomal genes and conjugative transposones, due to contamination by few other bacterial species in the sorted sample.

The further analysis of the single nucleotide polymorphism sites (SNPs) focused only on the toxin B gene which is specific to virulent *C. difficile* strains only.^7^ If other bacterial species are investigated by the same approach, a subset of genes specific to that particular species or its core genome, should be taken into account. In our case, the toxin B region (7,098 bp) contained three SNP variants. The obtained shotgun reads contained in the position 789,657 bp either the reference guanine or a novel adenine. In the position 789,660 bp it had either the reference thymine or a novel cytosine, and in the position 789,681 bp a cytosine (Figure 1). These nucleotide changes were synonymous substitutions. In order to find out whether these variants have been previously sequenced by other studies, the toxin B sequences containing the novel non-reference variants were aligned to the “nr” database of NCBI. The sequences matched with 100 % identity the sequence of the toxin B sequence types B07 and B08 isolated in China in September. 2014 ^16^ However, as no whole genome sequence is available for this Chinese isolate, we cannot conclude that the patient was infected by this particular strain.

## Future directions

The current high-throughput sequencing and cell sorting technologies provide new ways to study the natural genetic diversity of strains of selected pathogens that are present in a single patient at a very low abundance. Current improvements of the sequencing library preparation protocols allow the sequencing of samples containing only a few hundreds bacterial cells. The FACS-seq based approach presented here recovers multiple strains at their natural proportion and so facilitates epidemiological tracking of an outbreak. In addition, it may lead to discovery of novel pathogenic strains with unusual culture conditions requirements. Moreover, as there is no need to culture the cells of interest, this method can reduce diagnostic time, especially in cases of pathogens which produce colonies after several days or weeks of culture, such as *Legionella pneumophila* or *Mycobacterium tuberculosis*.^17^

In this pilot study, the proportion of *C. difficile* in the patient’s faecal microbiome was as low as 0.1%. We performed a shallow genome sequencing of 10,000 *C. difficile* cells, which was, however, sufficient for detection of possible genetic variants among the multiple strains in one patient’s sample. According to Li *et al*.,^18^ a reference genomes coverage of as few as 4x may be sufficient to detect differences among strains in metagenomes, however, the required minimal coverage may vary in distinct projects depending on the microbial community complexity and species similarity. After the presence of multiple SNPs in a sample are found in low genome coverage, the samples can be then sequenced more deeply to obtain a genome coverage of at least a hundred times. Such a high coverage would allow recovery of nearly complete genomes of multiple strains. According to Rinke *et al*.,^19^ a successful Illumina sequencing run can be achieved with as few as 100 femtograms of DNA (the DNA amount contained in 100–1000 bacterial cells) using a modified library preparation protocol. Confirmation of multiple strains in single patients by the FACS-seq approach may help to resolve links in an epidemiological network of disease outbreak detection obtained by common bioinformatic tools.^20^

However, it is important to note that despite a FACS-separated sample being enriched for the target pathogen species, it may also be contaminated by other bacterial species. Such contamination does not necessarily mean that the hybridization probes were non-specific. If the taxonomic composition of the contaminating species in the FACS-separated sample is the same as the taxonomic composition of the original non-FACS-separated sample, the source of the contamination may be the flow cytometer itself. The contamination of the FACS equipment is quite common and therefore, a rigorous cleaning procedure of the FACS equipment should be performed before the cell sorting.^21^ The reads belonging to the contaminating species may map weakly to the target pathogen genome forming peaks with extremely high coverage (as occurred in the present study in the 16S ribosomal gene regions and conjugative transposomes). The SNPs observed in these regions should not be considered for further analysis.

The potential contamination issues may be solved by metagenome binning. The shotgun reads are assembled and then mapped back to the assembled contigs. The characteristics (e.g. coverage, k-mer frequencies and GC content) of the contigs are compared and then clusters of similar contigs (so called bins) are formed.^22^ Bins belonging to the target pathogenic strains should be easily distinguished from the contaminating species. The genetic diversity of these strains may be assessed, including presence of plasmids or mobile genetic elements.

It is also important to mention that the fluorescently hybridized cells can be distributed by the flow cytometry equipment one by one into 384 well plates, so their whole genomes can be analyzed separately. In this approach, *Phi* polymerase adds random oligomers to the whole DNA molecule which results in amplification of the whole genome hundreds of times.^23^ Despite improving chemistry of the whole genome amplification, some regions of the bacterial genome can be accidentally omitted and the amplified sequences may contain polymerase proofreading errors. However, single-cell resolution can provide important information on large genomic differences between strains, such as mobile genetic elements.^24^ Therefore, single-cell genomic approach could be used for confirmation of unusual genomic rearrangements detected by sequencing of bulks of cells.
